# Adyan: automated annotating named entity recognition dataset for sorani kurdish language

**DOI:** 10.1016/j.dib.2025.111999

**Published:** 2025-08-21

**Authors:** Chovyan H. Wahid, Rebwar M. Nabi

**Affiliations:** a Computer Department, College of Science, Charmo University, Sulaymaniyah, Iraq; b Information Technology Department, Technical College of Informatics, Sulaimani Polytechnic University, Sulaymaniyah, Kurdistan Region, Iraq; c Information Technology Department, Kurdistan Technical Institute, Sulaymaniyah, Kurdistan Region, Iraq

**Keywords:** Low-resource languages, Named entity recognition, Sorani kurdish language, NLP

## Abstract

This paper introduces the first high-quality, automatically annotated Sorani Kurdish Named Entity Recognition (NER) dataset, addressing the lack of annotated resources for Kurdish, a low-resource language in Natural Language Processing (NLP). The corpus was collected from publicly available Kurdish news articles published in 2024 to ensure its relevance to contemporary language use. It spans a wide range of domains, including politics, economics, sports, health, culture, interviews, and technology, providing comprehensive coverage of named entities across various contexts. To ensure the trustworthiness of the content, the news articles were carefully selected from accredited Kurdish outlets. Annotation was performed using a lexicon-based approach, leveraging a pre-defined lexicon to maintain consistency and accuracy. The dataset was preprocessed as follows: entities were labeled using seed words from the pre-defined lexicon, and the BIO (Begin, Inside, Outside) tagging scheme was applied to ensure compatibility with widely used NER models. The dataset is available in TXT format (.txt), making it readily accessible and flexible for use in a variety of research applications. The Adyan dataset can be utilized for multiple NLP tasks, including NER, sentiment analysis, machine translation, and text classification. It is publicly released to support ongoing research and development in NLP for low-resource languages.

Specifications Table

**Table:** 

Subject	Computer Sciences
Specific subject area	Natural Language Processing, Named Entity Recognition
Type of data	Text format(.txt)
Data collection	The material was based on open access Kurdish news articles from 2024 and onwards. Articles being manually extracted, cleaning and normalization to be done by Python scripts. Named entities were labeled using a dictionary-based approach followed by BIO tagging. The dataset is stored in TXT files.
Data source location	https://www.rudaw.net/ https://www.ava.news/ https://www.nrttv.com/ https://www.kurdsat.tv/ckb
Data accessibility	Repository Name: Mendeley Data Data identification number: 10.17632/6gffcrcj75.2 Direct URL to data: https://data.mendeley.com/datasets/6gffcrcj75/2
Related research article	None

## 1 Value of the Data

• The **Adyan** dataset is a valuable digital resource for Sorani Kurdish, a low-resource language in NLP. It fills a critical gap by providing the first high-coverage, automatically annotated Named Entity Recognition (NER) corpus in this language.
• The dataset contains **654,404 labeled tokens** across **2300 articles** and supports 15 named entity types using the widely adopted BIO tagging format. Unlike previous Kurdish NER datasets that include only a few general categories such as *Person, Location*, and *Organization*, Adyan offers **fine-grained entity annotations** across multiple domains, including politics, economics, sports, religion, culture, interviews, and multimedia.
• This diversity ensures richer semantic coverage, enabling the development of more sophisticated NLP models and language technologies. The annotations were generated through a robust dictionary-based method and verified by native speakers, ensuring **high accuracy and consistency**. The dataset's structure and scale make it applicable to various NLP tasks such as NER, machine translation, sentiment analysis, and text classification.
• Furthermore, the dataset is openly available in a machine-readable TXT format and is accompanied by documentation to support reproducibility and usability by the broader research community.

## 2 Background

Kurdish Language is an Indo-European language which belongs to Northwestern Iranian sub-branch of the Indo-Iranian family of languages. The speakers of this language are predominantly the Kurds who live in parts of Turkey, Iraq, Iran, Syria and Armenia. Kurdish language has an approximate 30–40 million speakers, which makes it one of the most spoken languages in the Middle East, though, it does not have official state recognition in the majority of the regions where it is spoken. Although Kurdish has a great deal of linguistic and ethnical diversity, it has always been politically and academically oppressed which has affected its growth in fields like NLP [1].

Kurdish is not a monolithic language but a group of closely related dialects, each with distinct phonological, morphological, and syntactic features. The three main dialects are Sorani (Central Kurdish), primarily spoken in Iraq and Iran, written in the Perso-Arabic script, and recognized as an official language in the Kurdistan Region of Iraq [2]. Kurmanji (Northern Kurdish) is the most widely spoken dialect, used in Turkey, Syria, and parts of Iraq and Iran, and written in the Latin script [3]. Zazaki (also considered part of the Kurdish language group by some scholars) is spoken in eastern Turkey and differs significantly from Sorani and Kurmanji in grammar and phonology. These dialectal variations especially in script and morphology pose substantial challenges for NLP tasks such as standardization, named entity recognition, and cross-dialectal modeling [4].

Sorani Kurdish generally follows a Subject-Object-Verb (SOV) structure, although its word order can be flexible depending on emphasis and context. It primarily uses prepositions, and definiteness is typically marked morphologically. Sorani's rich inflectional morphology including case inflection, possessive suffixes, and pluralization adds complexity to linguistic processing and can lead to inconsistencies in annotation [5].

Despite linguistic diversity and rich culture, the Kurdish language has received little attention in the field of Natural Language Processing (NLP). This neglect is explicit within NLP tasks such as Named Entity Recognition (NER), where the absence of annotated corpora and standardized data sets has become an obstacle in the way of developing strong NLP models for Kurdish [6].

Existing NER datasets for Kurdish, such as KurdishBLARK [7], have contributed to resource development, but still exhibit several limitations:

• **Manual annotation:** These datasets rely heavily on human annotation, which is time-consuming, costly, and prone to inconsistencies—especially in low-resource languages like Kurdish [7].
• **Limited entity coverage:** Most existing resources categorize entities only under general types such as *Person, Location*, and *Organization*, limiting their applicability in more complex NLP tasks [7].
• **Insufficient data volume:** The relatively small corpus size and limited number of labeled entities restrict their usefulness for training robust NER models [7].
• **Disparities in entity definitions:** Variations in how entities are defined and labeled make it difficult to standardize training data across applications [7].

These limitations underscore the need for a larger, more comprehensive, and automatically annotated Kurdish NER dataset that supports fine-grained entity recognition across multiple domains.

Despite the existence of earlier Kurdish NER resources, most suffer from limitations in both size and entity diversity. These datasets typically consist of a small number of tokens, often collected from a single media source, and include only a limited range of entity types such as *Person, Location, Organization, Date*, and *Misc*. While such resources offer a valuable starting point for low-resource Kurdish NLP, their narrow entity coverage and lack of domain diversity make them unsuitable for more advanced and domain-heterogeneous NER applications.

The development of Kurdish Natural Language Processing (k-NLP), and more specifically Named Entity Recognition (NER), has been hindered by the scarcity of standardized and well-annotated data. The current resources are usually either not entities diverse enough, annotations with low quality or with poor scalability which is not appropriate for training an accurate and robust NER model. But these limitations are not limited to NER as they affect other tasks too, such as machine translation, sentiment analysis, and text classification, which in turn limits Kurds' proposition to technology [8,9].

To fill this gap, the current paper presents a high-quality lexicon-based NER resource for Sorani Kurdish. It uses a curated lexicon, during the annotation process, which boosts annotation consistency, increases entity recognition accuracy and better scalability [2]. The corpus covers different domains obtained from 2024 Persian news related websites, valuable content for NLP studies [3]. Furthermore, the dataset we provided can be helpful to develop NER systems and low-resource NLP applications and to enhance computational tools for the Kurdish language [1].

## 3 Data Description

This article introduces the Automatically Annotated Named Entity Recognition Dataset for Sorani Kurdish, which is based on 2,300news articles collected from five well-known Kurdish news websites:

• Rudaw (www.rudaw.net)
• Avanews (www.ava.news/)
• NRT (www.nrttv.com)
• Kurdsatnews (www.kurdsat.tv)

**Fig. 1:**
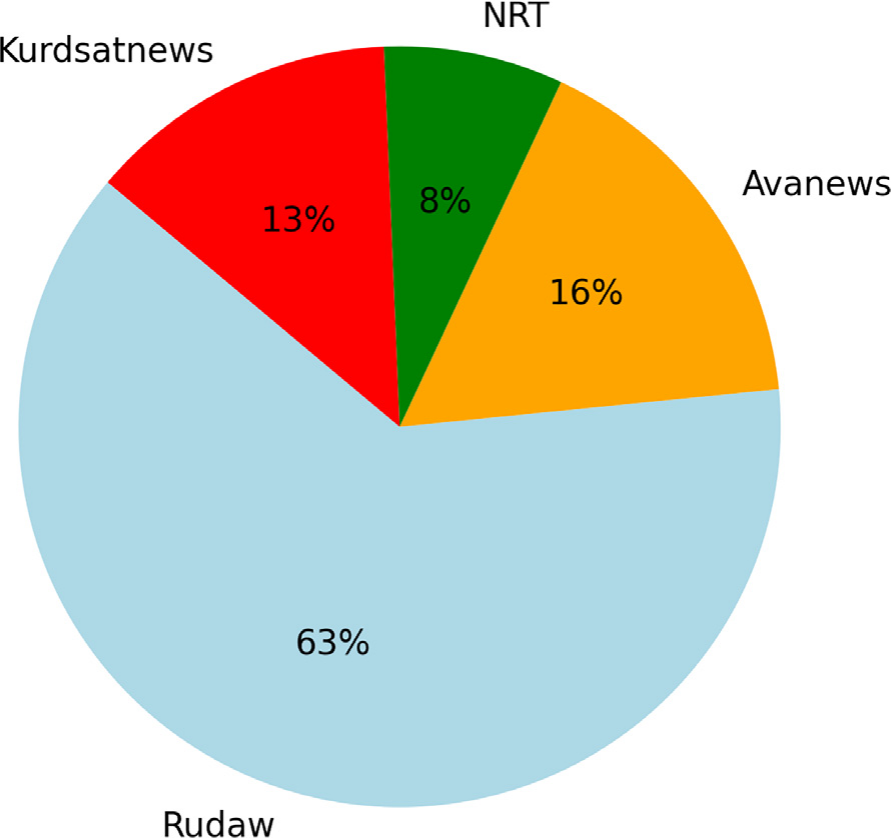
Distribution of News Articles by Source.

**Fig. 2:**
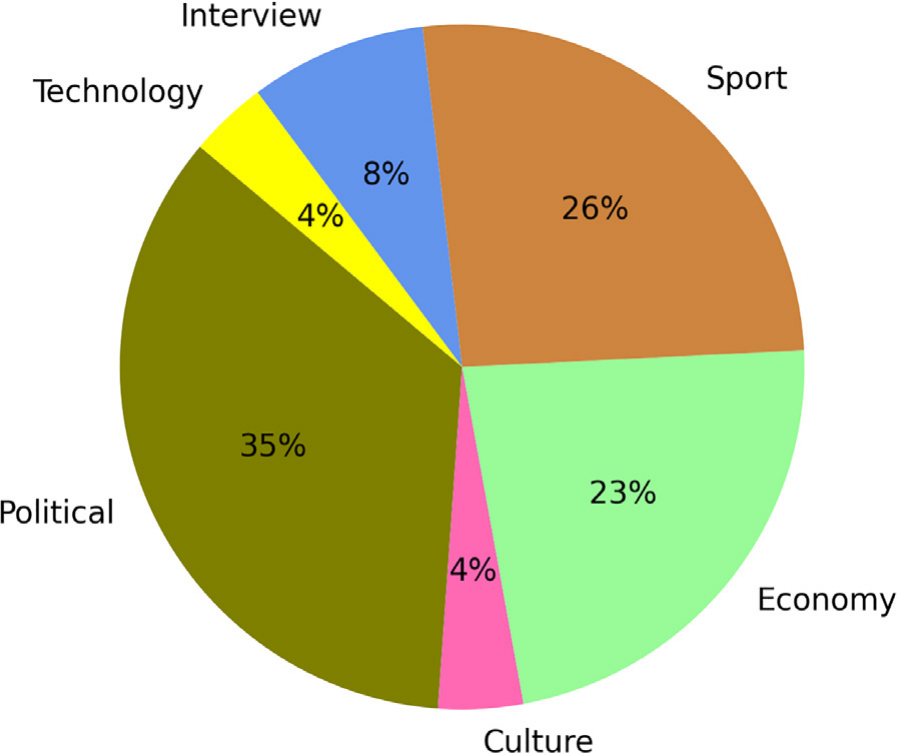
Distribution of News Articles by Domain**.**

These sources were chosen because they offer a well-rounded selection of current news in Sorani Kurdish. The corpus has contents classified under six main news topics (politics, economy, sports, culture, interview, technology), achieving a wide distribution of named entities in different context, as is illustrated in Fig. 1, Fig. 2.

The dataset was automatically annotated using a dictionary-based method with a fixed lexicon of 12,030 named entities distributed over 15 named entity types. So, this dictionary based labeling further improved the overfitting issue in the raw dataset, since the annotations are in high quality and consistent and it is easily scalable, which made the dataset become usable in the broader sense for Kurdish NLP applications.

### 3.1 Annotation process

• Automatic Matching: Words in the dataset were matched against predefined named entities.
• The dataset adopts the **BIO (Begin-Inside-Outside)** tagging scheme, which labels tokens as follows: **B-ENTITY** indicates the beginning of a named entity, **I-ENTITY** marks subsequent tokens within the same entity, and **O** designates tokens that are not part of any named entity. This scheme was chosen due to its widespread adoption in Named Entity Recognition (NER) research and its compatibility with a variety of established NLP toolkits. The BIO format is particularly advantageous for low-resource languages such as Sorani Kurdish, offering a balance of simplicity, clarity, and ease of integration into sequence labeling models. While alternative schemes like BILOU or BIOS provide more granular annotations, the BIO scheme was deemed both sufficient and efficient for representing entity structure in the context of this dataset, without introducing unnecessary complexity.
• Quality Control: A manual verification step was conducted on a subset of the dataset to correct misclassifications and improve consistency.

The dataset contains 654,404 labeled words categorized into 15 named entity types. The distribution of named entities within the predefined dictionary used for annotation is detailed in Table 1, highlighting the frequency of entities available for dictionary-based labeling.

**Table 1:** Named Entity Types in the Dictionary.

Entity Type	Count
Country	223
Season	4
Days	13
Month	30
Currency	156
Sport	432
Persons	9120
Locations	1247
Organization	329
Position	154
Political Party	78
Religion	19
MISC	128
Event	81
Equipment	25
Total Named Entities in Dictionary	12,030

The dataset, structured in TXT format, follows the BIO tagging scheme, where each word is paired with its respective named entity label Table 2 presents an example annotation:

**Table 2:** Example of Annotated Named Entities in the Dataset.

## 4 Experimental Design, Materials and Methods

**Fig. 3:**
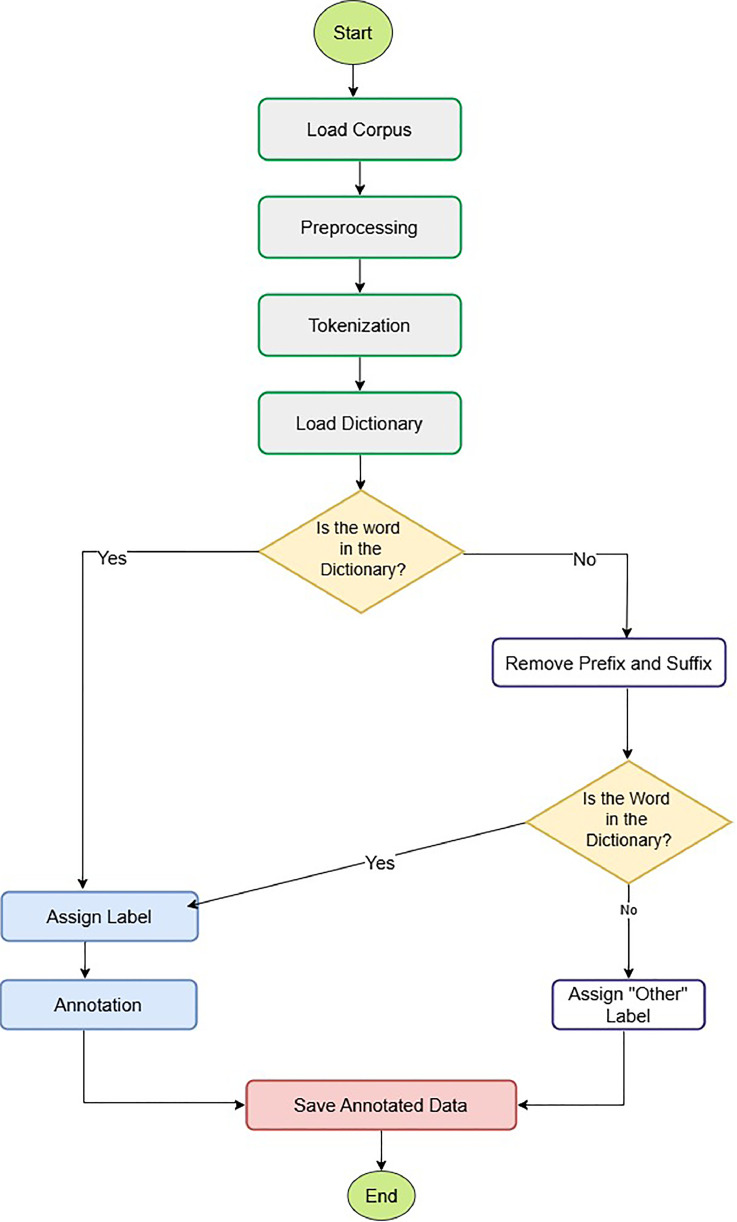
The Automated Annotation Workflow for Kurdish NER**.**

The building process and the annotations provided for the manually validated Adyan dataset, Automatically Annotated NER dataset for Sorani Kurdish are described in this section. Prior to semantic annotation, the data was preprocessed, tokenized, annotated on dictionary-based concept and validated to guarantee high accuracy, consistency and scalability. The entire annotation process applied in this dataset is presented in Fig. 3.

### 4.1 Corpus loading

Corpus Loading Kurdish news articles are used as a dataset that is being will be uploaded to system for implementation.

### 4.2 Pre-processing

The raw text has been subjected to various pre-processing steps in order to improve the data quality and consistency

A. Text Cleaning & Normalization:
  ○ Removed unnecessary characters, symbols, and punctuation inconsistencies.
  ○ Standardized punctuation rules and unified spelling variations in Sorani Kurdish.
B. Whitespace & Redundancy Removal:
  ○ Eliminated extra spaces and redundant text elements.
C. Duplicate Detection & Removal:
  ○ Identified and removed duplicate articles to maintain dataset uniqueness.

To illustrate the preprocessing impact, an example is provided:

Before Processing:



*“Lavrov: The* Syrian *authorities have not asked us to change our agreement regarding our stay in that country. Digital Rudaw, Sergey Lavrov, Russian Foreign Minister says,”*

After Processing:



*“Lavrov The Syrian authorities have not asked us to change our agreement regarding our stay in that country Digital Rudaw Sergey Lavrov Russian Foreign Minister says”*

### 4.3 Tokenization

Each article was segmented into sentences and subsequently tokenized into individual words. This step was crucial for word-level entity recognition and provided structured text units for annotation.

### 4.4 Dictionary-based annotation

A predefined lexicon containing 12,030 named entities across 15 categories was used for automatic annotation. Each token was matched against the lexicon, and entity labels were assigned accordingly.

• If a match was found, the corresponding entity label was assigned.
• If no match was found, the token was stripped of prefixes and suffixes and rechecked.
• Tokens that remained unmatched were labeled as "Other" (non-entity).

To standardize the annotation, the BIO tagging scheme was applied, where tokens are labeled as B-Entity, I-Entity, or O, as introduced earlier.

The choice of a dictionary-based annotation approach was driven by the low-resource nature of the Sorani Kurdish language. Currently, there are no large-scale annotated corpora or robust pre-trained models for Sorani, which makes machine learning-based annotation infeasible without significant manual effort. A dictionary-driven method enables consistent and scalable annotation using a predefined lexicon, ensuring high precision and reproducibility. This approach is especially effective in low-resource scenarios where maintaining annotation quality is critical despite limited computational and data resources.

### 4.5 Validation & refinement

The final annotated dataset is stored in BIO format, ensuring structured, machine readable annotations for Kurdish NLP research.

To ensure high-quality annotation, a manual validation step was performed on a random subset of 30 % of the dataset. The validation included:

1. Error Review
  • Manually identifying and correcting misclassified entities (e.g., incorrect entity types or missed entities).
2. Pattern Analysis:
  • Reviewing common misclassification patterns (e.g., ambiguous entities or partial matches) to refine the dictionary-based annotation rules.
3. Quality Assurance:
  • Verifying annotation consistency to meet linguistic and computational standards for Named Entity Recognition (NER) tasks.

This revised annotation and validation approach enhances accuracy, scalability, and usability for NLP applications.

Furthermore, to ensure the accuracy and quality of the labeled data, the automatically generated annotations were subsequently verified and refined by two independent language experts with expertise in the target language, in which their names written in the acknowledgment section. The annotations were carefully reviewed, and any discrepancies were resolved through mutual agreement between the annotators.

Inter-Annotator Agreement (IAA) was assessed using Cohen’s Kappa (κ), which is a robust measure of agreement between two annotators while accounting for agreement occurring by chance. The Cohen’s Kappa value for this dataset was calculated to be κ = 0.92, which is considered an “almost perfect” agreement according to standard interpretation (κ > 0.80). This high IAA score not only validates the quality and reliability of the annotated dataset but also demonstrates the effectiveness of the initial automated labeling process when combined with expert verification.

### 4.6 Algorithm: annotate corpus

The annotation of named entities in the Dataset is performed automatically using a previous lexicon-based approach, aimed at making the labelling process more efficient and consistent. The dataset is preprocessed and tokenized, matched with entities and labelling by the BIO format to guarantee high-quality annotations. Our annotation procedure is summarized by Algorithm 1.

**Algorithm 1:** AnnotateCorpus.

**Input**: *KurdishNewsDataset* (Kurdish news articles) **Output**: Labeled dataset in BIO format 1. **Corpus Loading**: 1.1. Load *KurdishNewsDataset* into the system. 2. **Preprocessing**: For each article in KurdishNewsDataset, do: a. Remove extraneous characters. b. Standardize punctuation. c. Apply orthographic normalization (correct spelling and formatting inconsistencies). d. Remove redundant spaces and unwanted symbols. 3. **Tokenization**: For each pre-processed article, do: a. Segment the text into individual tokens (words). 4. **Dictionary Matching**: For each token in the article, do: a. Check token against a predefined lexicon of named entities. i. If a match is found: - Assign the corresponding entity label to the token. ii. If no match is found: - Remove prefixes and suffixes from the token. - Recheck the token in the lexicon. - If a match is found after rechecking: * Assign the corresponding entity label. - Otherwise: * Label the token as **'O' (non-entity)**. 5. **Annotation Saving**: 5.1. Save the labeled dataset in **BIO format** (Begin, Inside, Outside tags) to ensure structured and standardized annotations for further NLP tasks. **End Algorithm**

## Limitations and challenges

The dataset faces certain limitations that require further refinement. The dialect-centric focus on the Sorani dialect is one limitation, as it may impact its usefulness with other Kurdish dialects like Kurmanji or Zazaki. Another limitation is named entity disambiguation where any difference in spelling or transliteration can create issues in annotations. There is also the issue of temporal coverage since the only source material comes from 2024 news articles onwards, which means all pre-2024 articles and their linguistic variations have been ignored.

Beyond these setbacks, there are other obstacles related to creating a precise Kurdish NER dataset. It is harder to standardize because there are multiple languages in Kurdish, including Sorani, Kurmanji, or Zizaki, all with their own unique dialects structures and even sets of rules for writing [6]. Additionally, removing the head casing makes it even more difficult for people speaking Kurdish, compared to English. It also makes entity recognition and classification much more difficult [10]. It also poses problems for the use of Kurdish that uses two scripts, the Sorani is in Perso-Arabic and Kurmanji is in Latin.

## Ethics Statement

The authors confirm that this work complies with the ethical requirements for publication in Data in Brief. The dataset was compiled from publicly available news articles, and no personal or sensitive data were used. No human subjects or animal experiments were involved in this research.

## Credit Author Statement

**Chovian H. Wahid:** Collected and processed the dataset, implemented dictionary-based labeling, conducted entity annotation, performed data analysis, and wrote the manuscript. **Rebwar M. Nabi:** Supervised the research, provided guidance, and reviewed the manuscript.

## Data Availability

Mendeley DataAdyan: A High-Quality Automated NER Dataset for Sorani Kurdish (Original data).
